# *CNKSR2*-related neurodevelopmental and epilepsy disorder: a cohort of 13 new families and literature review indicating a predominance of loss of function pathogenic variants

**DOI:** 10.1186/s12920-021-01033-7

**Published:** 2021-07-15

**Authors:** Leigh Ann Higa, Jennifer Wardley, Christopher Wardley, Susan Singh, Timothy Foster, Joseph J. Shen

**Affiliations:** 1 Department of Pediatrics, Community Regional Medical Center, Fresno, CA USA; 2 CNKSR2 Family Support Group, Rochdale, Lancashire UK; 3 CNKSR2 Family Support Group, Sanger, CA USA; 4 Division of Pediatric Neurology, Department of Pediatrics, UCSF Fresno, Fresno, CA USA; 5 Division of Genetics, Department of Pediatrics, UCSF Fresno, Fresno, CA USA; 6 Division of Genomic Medicine, Department of Pediatrics, MIND Institute, University of California, Davis, 2825 50th Street, Sacramento, CA 95817 USA

**Keywords:** CNKSR2, CNK2, Epilepsy, ESES, CSWS, Developmental delay

## Abstract

**Background:**

Pathogenic variants in connector enhancer of kinase suppressor of Ras-2 (*CNKSR2*) located on the X chromosome (Xp22.12) lead to a disorder characterized by developmental delay and a characteristic seizure phenotype. To date, 20 affected males representing 13 different pathogenic variants have been published.

**Case presentation:**

We identified an 8-year-old male with seizures, abnormal electroencephalogram (EEG) with epileptiform abnormalities in the right hemisphere, and developmental delay with notable loss of speech following seizure onset. Additional concerns include multiple nighttime awakenings, hyperactivity, and autism spectrum disorder. Genetic testing identified a de novo pathogenic nonsense variant in *CNKSR2.*

Through an active family support group, an additional 12 males are described, each harboring a different *CNKSR2* variant. The clinical presentation and natural history consistently show early developmental delay, sleep disturbances, and seizure onset in childhood that is initially intractable but later becomes better controlled. Virtually all of the pathogenic variants are predicted to be loss of function, including genomic deletions, nonsense variants, splice site mutations, and small insertions or deletions.

**Conclusions:**

This expanded knowledge, combined with functional studies and work with animal models currently underway, will enable a better understanding and improved ability to care for individuals with *CNKSR2*-related neurodevelopmental and epilepsy disorder.

**Supplementary Information:**

The online version contains supplementary material available at 10.1186/s12920-021-01033-7.

## Background

Connector enhancer of kinase suppressor of Ras-2 (*CNKSR2*, MIM 300724), with alternative designations in the literature including CNK2 and MAGUIN [18] was first described as Cnk in *Drosophila melanogaster* as a component of the Ras signaling pathway [16]. It is expressed predominantly in the brain ([12], Human Protein Atlas) and functions as a scaffold protein within the postsynaptic density mediating numerous signaling pathways and neuronal morphogenesis, consistent with postulated roles in membrane and cytoskeletal remodeling [3, 4, 8, 10, 11, 19].

Since the initial characterization of a boy affected with a pathogenic variant in *CNKSR2* by Houge et al. [7] (mental retardation, X-linked, Houge type (MRXSHG), MIM 301008), there have been a total of 20 affected males described in the literature, all with a consistent clinical phenotype of intellectual disability, developmental delay, and epilepsy [1, 2, 5, 6, 15, 17, 20]. Early developmental progress may be normal in rare instances, but global delays are observed consistently after the onset of seizures. Sleep disturbances are common as well. Seizures in *CNKSR2*-related neurodevelopmental and epilepsy disorder are typically difficult to control, and the electroencephalographic findings are fairly characteristic and categorized as encephalopathy with status epilepticus during slow wave sleep (ESES) as well as continuous spike waves in sleep (CSWS).

Approximately half of the known instances of *CNKSR2*-related disease are de novo in origin, and of the maternally transmitted cases, the majority of carrier mothers are neurologically without concerns. However, some females heterozygous for a *CNKSR2* pathogenic variant have intellectual disability and seizures [2, 5, 6, 14, 20].

A total of 13 different variants in these 20 males have been reported and, interestingly, all are expected to be loss of function, whether from genomic deletions encompassing the entire *CNKSR2* locus, nonsense variants, intronic variants resulting in aberrant splicing, or small insertions/deletions predicted to lead to a frameshift and downstream premature protein truncation.

We diagnosed a child with *CNKSR2*-related neurodevelopmental and epilepsy disorder, and in conjunction with a family-driven effort through the international CNKSR2 Family Support Group also present a cohort of an additional 12 affected males from different families each harboring unique and previously undescribed pathogenic variants. The Case Report section will describe the proband as family 1, and clinical descriptions of families 2 through 13 will be in Additional file 1. The parents or legal guardians contributed the clinical and molecular genetic information and provided written informed consent for research participation in this study approved by the Institutional Review Board at Community Regional Medical Center. All individuals presented with seizures and, with the exception of one child (family 5), exhibited intellectual disability/developmental delay prior to seizure onset.

## Case presentation

The proband for family 1 is an 8-year-old male born at term weighing 3912 g after an unremarkable pregnancy and delivery. Family medical history is without close relatives with similar concerns and the parents deny consanguinity. Early developmental milestones include sitting at 7 months and walking at 17 months. At 2.5 years of age he spoke a few words, but then after the onset of seizures at age 4, he became non-verbal. Slowly over the course of the intervening years he has regained some language and currently says about 10 words. Fine motor skills include operating a tablet computer and at least drawing a circle. He has been diagnosed with autism spectrum disorder. His first seizure was at 4 years of age with a prolonged staring episode. The next seizure occurred several months later but then became more frequent and harder to control. Multiple electroencephalograms (EEGs) have been read as epileptiform abnormalities in the right hemisphere, patterns consistent with Landau-Kleffner, and spiking occurring at night. He had been started on antiepileptic treatment (levetiracetam) without success, and now is somewhat better controlled on cannabidiol (CBD) oil with continued titration of the CBD:tetrahydrocannabinol (THC) formulation. Sleep is significantly disrupted with him generally being a light sleeper and having absence, tonic–clonic, and unusual laughing episodes (gelastic) seizures mainly at night. Melatonin has only slightly helped in the past, but now CBD oil may be of greater benefit. There are also nocturnal episodes of nonresponsiveness. Generally at baseline he is quite agitated and hyperactive. No dysmorphic features were noted on physical examination. Both head computed tomography (CT) and brain magnetic resonance imaging (MRI) are without findings of note.

A diagnosis of *CNKSR2*-related neurodevelopmental and epilepsy disorder was made via genetic testing. Clinical whole exome sequencing (InVitae Corporation, San Francisco, CA, USA) identified a novel nonsense variant in *CNKSR2*, NC_000023.10: g.21627392T>G NM_014927.5: c.2349T>G, p.(Tyr783*). Subsequent testing of his mother determined that this *CNKSR2* variant is not maternally inherited.

### Overview of cohort

Our cohort includes 13 males from 13 families, with the case we diagnosed as family 1. The age range is 3 years to 20 years, and the average age of our cohort is 8.7 years. Birth history consists of full term delivery for 11/13 males (85%), and preterm delivery at 36 weeks and 36 weeks 6 days, for two males, respectively (15%).

The two clinical characteristics observed universally in our cohort are seizures and intellectual disability (Table 1). The age of seizure onset ranges from 4 days of age to 4 years, with a mean age of 20.6 months. Seizure type is variable, and includes absence, atonic, tonic clonic, complex partial, focal, gelastic, dacrystic seizures. Face and hand twitching have also been reported. Six of 13 males (46.1%) had EEG documenting electrical status epilepticus during sleep, also known as continuous spikes and waves during sleep (ESES/CSWS, families 2, 6, 8, 10, 11, 13). Other EEG abnormalities reported include, but are not limited to, prolonged seizures, focal epilepsy, and clusters of seizures during day and night (Additional file 1). Four males were diagnosed with Landau Kleffner (4/13 or 30.8%; family 7, 8, 10, 13) and one has a diagnosis of Lennox Gastaut (1/13 or 7.7%; family 6).

**Table 1:** Clinical and molecular summary of *CNKSR2*-related neurodevelopmental and epilepsy disorder

Family	Age (years)	Age of seizure onset	Intellectual disability?	Maternal origin?	Variant^a^	Source
1	8	4y	Y^c^	N	c.2349T>G, p.(Tyr783*)	This study
2	21	15m^b^	Y	Y	c.1537C > T, p.(Pro513Ser)^e^	This study
3	5	2y	Y^c^	N	c.1988_1989del, p.(Arg663Asnfs*2)	This study
4	10	6m	Y	N	c.1653_1656del, p.(Asn551Lysfs*4)	This study
5	9	4y	N → Y^d^	Y	c.2545C > T, p.(Arg849*)	This study
6	8	7d	Y^c^	N	c.2145 + 1G > A	This study
7	9	8m	Y^c^	Y	arr [hg19] Xp22.12 (21,278,397–21,678,707) × 0	This study
8	7	2y	Y	N	c.1198C > T, p.(Arg400*)	This study
9	9	4d	Y^c^	N	c.2044 + 2 T > A	This study
10	5	2y	Y^c^	N	c.520-1G > A	This study
11	11	23m	Y	N	c.2005del, p.(Ala669Glnfs*48)	This study
12	3	2y	Y^c^	N	c.1905-2A > G	This study
13	8	2y	Y^c^	N	c.2026_2027del, p.(Arg676Aspfs*2)	This study
14	7	2y	Y	Y	arr [hg19] Xp22.12 (21,322,029–21,678,137) × 0	Aypar et al. [1]
15	5	2.5y	Y	Y	arr [hg19] Xp22.12 (21,375,312–21,609,484 × 0	Houge et al. [7], Vaags et al. [17] pt 3
16	6	2y	Y^c^	Y	arr [hg19] Xp22.12 (20,297,696–21,471,387) × 0	Vaags et al. [17] pt 1
	8	27m	Y^c^	Y		Vaags et al. [17] pt 2
17	12	8d	Y^c^	Y	arr [hg19] Xp22.12 (21,193,947–21,707,169) × 0	Vaags et al. [17] pt 4
	13	N/A	Y^c^	Y		Vaags et al. [17] pt 5
18	56	?^b^	Y^c^	Y	g.21458832_21458833ins, p.(Asp152Argfs*8)	Vaags et al. [17] pt 6, Hu et al. [8], family P180
	58	?^b^	Y^c^	Y		Vaags et al. [17] pt 7, Hu et al. [8], family P180
	62	?^b^	Y^c^	Y		Vaags et al. [17] pt 8, Hu et al. [8], family P180
19	18	3.5y	Y^c^	Y	c.2341C > T, p.(Arg712*)	Damiano et al. [5]
	12	3.5y	Y	Y		Damiano et al. [5] brother
	?	3.5y^b^	Y	?		Damiano et al. [5] maternal uncle^f^
20	9	2y	Y	N	c.2185C > T, p.(Arg729*)	Sun et al. [15]
21	6	2y	Y	N	c.2024_2027del, p.(Glu657Glyfs*41)	Bonardi et al. [2] pt 1
22	21	3y^b^	N → Y^d^	N	c.246_247del, p.(Thr83Lysfs*30)	Bonardi et al. [2] pt 2
23	12	4y	Y^c^	Y (mosaic)	c.457_461del, p.(Tyr153Serfs*5)	Bonardi et al. [2] pt 3
24	10	3y	Y^c^	N	arr [hg19] Xp22.12 (21,609,392–21,619,786) × 0	Bonardi et al. [2] pt 5
25	5	4y	Y	Y	arr [hg19] Xp22.12 (21,606,698–21,616,207) × 0	Daoqi et al. [6]
	“Younger”	3y	Y	Y		Daoqi et al. [6] brother
26	6	N/A	Y	Y	c.1904 + 1G > A	Zhang et al. [20]
Females						
	–	N/A	N	?	c.1537C > T, p.(Pro513Ser)	Mother in family 2
	–	N/A	N	?	c.2545C > T, p.(Arg849*)	Mother in family 5
	–	N/A	N	?	arr [hg19] Xp22.12 (21,278,397–21,678,707) × 1	Mother in family 7
	–	N/A	N	?	arr [hg19] Xp22.12 (21,322,029–21,678,137) × 1	Mother in family 14
	–	N/A	N	N	arr [hg19] Xp22.12 (21,375,312–21,609,484) × 1	Mother in family 15
	–	N/A	N	?	arr [hg19] Xp22.12 (20,297,696–21,471,387) × 1	Mother in family 16
	16	6y	Y	Y	c.2341C > T, p.(Arg712*)	Sister in family 19
	?	febrile	N	N		Mother in family 19
27	7	5y	Y	N	c.2304G > A, (p.Trp768*)	Polla et al. [14]
28	41	6y^b^	N → Y^d^	?	arr [hg19] Xp22.12 (21,523,673–21,558,329) × 1	Bonardi et al. [2], Lesca et al. [9] pt 4
	?	Junior high	Y	N	arr [hg19] Xp22.12 (21,606,698–21,616,207) × 1	Mother in family 25
	33	N/A	?	N	c.1904 + 1G > A	Mother in family 26

*d* Days, *m* months, *y* years, *N/A* not applicable, *pt* patient

^a^In this cohort, transcript NM_014927 (GRCh37p13) confirmed for families 1, 2, 4, 5, 6, 7, 8, 11, 12, 13

^b^Seizures stopped in pre-teen years

^c^Minimal-to-no speech

^d^Developmental delay manifested after onset of seizures

^e^Classified by laboratory as variant of uncertain significance

^f^Not tested for familial *CNKSR2* pathogenic variant, but neurological and seizure phenotype matches that seen in the proband

Additional neurodevelopmental diagnoses reported in more than one individual include autism (8/13 or 26.7%, families 1, 3, 4, 5, 7, 9, 12, 13) and attention deficit hyperactivity disorder (3/13 or 24%; families 1, 7, 13). Further clinical information for the probands in families 2 through 13 are provided in Additional file 1.

Twelve (92.3%) of the *CNKSR2* variants in this cohort were classified as likely pathogenic or pathogenic, with the remaining one (missense variant in family 2) a variant of uncertain significance. As shown in Table 1, insertion/deletion (in/del) variants and splicing variants were each seen in 4/13 (30.8%) of affected individuals, nonsense variants were noted in three individuals (23.1%), and one male harbored a contiguous deletion (7.7%). All mothers completed genetic testing to establish carrier status. Three mothers were found to be carriers, for an incidence of 23.1%, and none have had a history of seizures or neurodevelopmental concerns.

## Discussion and conclusions

The neurodevelopmental phenotype and seizure types exhibited by the affected males in this cohort was consistent with and further validated the patterns and characteristics observed in the previously published cases of *CNKSR2-*related neurodevelopmental and epilepsy disorder (Table 1). The degree of developmental delay can be severe and disproportionately affect language skills, with 8 of the 13 (62%) of the individuals in our cohort with very few or no words, similar to that described in the published individuals with at least 10 of 20 reported to have no or very minimal speech. Significant absence and generalized tonic–clonic seizures are observed, oftentimes both in the same individuals, which generally are difficult to control with anti-epileptic medication. Interestingly, with most if not all of the older affected individuals reported in the literature, despite the medically intractable nature of their epilepsy during early childhood, there appears to be resolution of the seizures and weaning off of anti-epileptic medication in the pre-teen years. The sole adult in our cohort (family 2) matched this natural history pattern with complete cessation of his seizures and no further medication by 10 years of age.

Additional characteristics were noted which expand our understanding of the neurobehavioral and clinical phenotype. Sleep disturbances were reported by the parents to be present in 8 of the 13 (62%) affected males in this cohort, as first appreciated in Vaags et al. [17]. Significant hyperactivity was present in 6 (46%) as reported by the parents. Four (31%) of the affected males had significant feeding issues necessitating the use of a feeding tube in early childhood, but in later years there was not consistently observed failure to thrive in them or the others in the cohort (data not shown).

Another commonality extending from the published cases of *CNKSR2-*related neurodevelopmental and epilepsy disorder is the type of pathogenic variants detected—loss of function continues to be the predominant pathogenic molecular mechanism of disease. As shown in Table 1, in our cohort 3 nonsense variants, 1 genomic deletion encompassing the *CNKSR2* locus, 4 small indels predicted to lead to a frameshift and downstream stop codon, and 4 splice site variants, have been identified; only family 2 harbored a missense variant classified as a variant of uncertain significance. This observation that virtually all pathogenic *CNKSR2* variants are loss of function is consistent with this gene’s probability of being loss-of-function intolerant (pLI) score of 1.0 and loss-of-function observed/expected upper bound fraction (LOEUF) score of 0.185 (gnomAD v2.1.1).

Of the 13 affected males in our cohort, ten represent de novo cases, while in three the variants are maternally inherited. Recent articles have indicated that females harboring heterozygous *CNKSR2* pathogenic variants may have seizures, with some also manifesting neurodevelopmental concerns (family 26, [14]; family 27, [2]; mother and sister of family 19, [5]; mother of family 25, [6]; mother of family 26, [20]). However, for another six mothers (three mothers in this cohort, family 14, [1]; family 15, [7]; family 16, [17], there were no reported seizures or intellectual disability. Skewed X-inactivation may be the underlying reason for this observed difference in penetrance (85:15 in the mother of family 25, [6] and 20:80 in family 26, [14], both with neurodevelopmental concerns, while reported to be 57:43 for the mother reported to have no neurological issues of family 15, [7]).

The size of our cohort is one of the key strengths of this report, permits us to confirm and expand upon the previously reported clinical phenotype and, importantly, corroborates loss of function of CNKSR2 as the principal molecular pathogenic mechanism of disease. The main limitation in this cohort is the variable amount of information for each individual, due to the fact that the families are located on different continents and obtaining full clinical and molecular documentation is a logistical challenge. To address this, we have focused our clinical data collection on the key phenotypic features of developmental delay/intellectual disability, seizures, and sleep disturbance. Accordingly, we acknowledge that novel features of *CNKSR2*-related neurodevelopmental and epilepsy disorder may remain unrecognized at this time, and await further characterization.

In conclusion, we report on a cohort of affected individuals with *CNKSR2-*related neurodevelopmental and epilepsy disorder that doubles the number of known pathogenic variants and corroborates CNKSR2 loss of function as the predominant molecular mechanism of disease. The neurodevelopmental and clinical phenotype is consistent with that previously reported in the literature with prominent features of intellectual disability and seizures. Our large cohort further bolsters and extends previous observations including noting significant sleep disturbances, hyperactivity, and some early feeding concerns. Approximately half of all known females heterozygous for a pathogenic *CNKSR2* variant have a similar but sometimes milder neurological phenotype, but the other half are without neurodevelopmental concerns or seizures. An under-recognized aspect of the epilepsy appears to be that it decreases in severity during childhood and, in almost all instances, resolves in the pre-teen years; knowing this natural history may be useful in guiding seizure management. Our hope is that understanding the complete phenotype of *CNKSR2-*related neurodevelopmental and epilepsy disorder will bring us closer towards the ultimate goal of investigating treatment options for individuals affected by this disease.

## Supplementary Information

**Additional file 1**. Supplemental Clinical Information for Families 2 through 13

## Data Availability

The sequence datasets generated during the current study are not publicly available because it is possible that individual privacy could be compromised. Data are available from the corresponding author upon reasonable request.

## Abbreviations

CNKSR2: Connector enhancer of kinase suppressor of Ras-2
EEG: Electroencephalogram
ESES: Status epilepticus during slow wave sleep
CSWS: Continuous spike waves in sleep
EEG: Electroencephalogram
CBD: Cannabidiol
THC: Tetrahydrocannabinol
CT: Computed tomography
MRI: Magnetic resonance imaging
in/del: Insertion/deletion
pLI: Probability of being loss-of-function intolerant
LOEUF: Loss-of-function observed/expected upper bound fraction
