# Supplementary material for: CNKSR2-related neurodevelopmental and epilepsy disorder: a cohort of 13 new families and literature review indicating a predominance of loss of function pathogenic variants
Source: BMC Med Genomics. 2021 Jul 15;14:186. doi: 10.1186/s12920-021-01033-7 (PMC8281706; doi:10.1186/s12920-021-01033-7)
Supplement: Supplementary file 1 — Additional file 1. Supplemental Clinical Information for Families 2 through 13 [file 12920_2021_1033_MOESM1_ESM.docx]

**Supplemental Clinical Information for Families 2 through 13**

**Family 2**

He is a 21-year-old male born full term weighing 3827 grams (38.2%ile), with seizure onset at 15 months of age. Early developmental milestones included sitting at 6 months and walking at 18 months. Verbal skills began at 3 years of age predominantly consisting of echolalia and some barely intelligible words, that then slowly advanced to understandable words by 10 years of age. Current developmental level is considered to be that of a 10-year-old. Family medical history is without close relatives with similar concerns. Seizures were of the tonic-clonic type which were initially poorly controlled leading to VNS placement. EEG showed spike and slow waves during sleep. He is still on clobazam although he has been seizure-free since 10 years of age. Sleep pattern is poor and he continues to take melatonin. Feeding has always been difficult, with a feeding tube placed at 2 years of age. Now as an adult, he is noted to be smaller than expected compared to parental heights and sizes (height 165.1 cm, with midparental height of 184.3 cm). He was briefly examined when visiting other CNKSR2 Family Support Group members, and not felt to be dysmorphic. Hyperactivity is not reported by his parents.

**Family 3**

He is a 5-year-old male born at term weighing 3250 grams (11.1%ile). His first seizure occurred at 2 years of age. His seizures mainly occur at night often starting with the absence type, but he does have tonic-clonic seizures as well. EEG demonstrated spiking; brain MRI in the past was reported as normal. He is on levetiracetam. He has global delays with recent evaluations assessing him at 18 months of age developmentally. He is not toilet trained. Prior to onset of seizures, he spoke a few words but now is non-verbal. His sleep is “not good” per his parents and he is on melatonin with some benefit. Parents state that he is hyperactive overall.

**Family 4**

He is a 10-year-old male born full term weighing 4026 grams (52%ile). Seizure onset was at 6 months and described as tonic-clonic and complex partial seizures. EEGs were read as non-focal epilepsy. His seizures have been difficult to control even after VNS implantation and on lacosamine, zonisamide, and CBD oil. He has significant intellectual disability with very limited verbal skills consisting of a few single words. He is not toilet trained and still is in diapers. There are no reported eating concerns and in fact his parents report that he eats more than expected for his age. His sleep is poor and he has been prescribed clonidine. Hyperactivity is not reported by his parents.

**Family 5**

He is an 9-year-old male who was born full term weighing 2930 grams (4.6%ile). The first episode concerning for seizures occurred at age 4 years. Currently his seizures are of the tonic-clonic as well as the absence type. Brain MRI in the past was read as normal. His most recent medications include valproate, clobazam, brivavact, midazolam. Developmentally, he walked at 12 months, and started talking between 10-14 months. Currently, he speaks in sentences, recognizes letters and numbers, and can somewhat count. However, he cannot read. He attends a school for children with learning disabilities, and behavior is notable for hyperactivity.

**Family 6**

He is an 8-year-old male who was born full term weighing 3827 grams (37.8%ile). Seizures began at 1 week old. EEG was initially normal, but now exhibits various abnormalities, including continuous spike and slow waves during sleep (CSWS). Currently he has many seizure types including myoclonic, gelastic, grand mal, drop attacks, and absence seizures. A VNS has been placed. Most recent medications used include topiramate, clobazam, banzel, zonisamide, lamotrigine, Vyvanse, epidiolex. He has developmental delay and started walking at 24 months. When last assessed at 6 years of age, he was nonverbal, understood and used a few signs, and was not toilet trained. A feeding tube was required due to failure to thrive. Current behavior is notable for hyperactivity.

**Family 7**

He is a 9-year-old male who was born full term weighing 2788 grams (2.93%ile). He had his first seizure at 8 months of the tonic-clonic type, with his EEG demonstrating clusters of seizures during both day and night. A VNS has been placed. Most recently, he was on clonazepam, oral steroids, and diastat for seizure control. A brain MRI in the past was reported to be abnormal, but additional details are not known. He walked at age 2 years, and when last assessed at age 8 was not toilet trained and still used a sippy cup. He is minimally verbal, and has only a few single words. His parents report his sleep to be very restless which is somewhat helped by melatonin. He required a feeding tube at 3 years of age. Additional diagnoses for him include hyperactivity and autism.

**Family 8**

He is a 7-year-old male who was born at 36 weeks weighing 2353 grams (0.66%ile). Seizures started at age 2, and he has had tonic-clonic, absence, focal, gelastic, and dacrystic seizures. His seizures tend to occur during sleep and generally are difficult to control. Most recent medications were clobazam, Depakote, and Ativan. His EEG abnormalities have been diagnosed officially as electrical status epilepticus during slow wave sleep (ESES). Brain MRI in the past was read as normal. He has global developmental delays. At 6.5 years of age, he was noted to have 50 words, was learning sign language, and started to feed himself some foods and drink from a cup. His parents report him to be hyperactive.

**Family 9**

He is a 9-year-old male who was born full term weighing 3289 grams (12.3%ile). Seizures began at 4 days old, and he has had status epilepticus, absence, tonic-clonic, focal, and gelastic seizures. EEGs were initially normal, but later demonstrated abnormal frontal lobe activity. Most recent medications include valproate, clobazam, and lacosamide. Developmentally, he sat independently at 7 months and walked at age 2, although currently he still has a wide gait and falls frequently. He is nonverbal but is able to utilize simple signs to communicate. Feeding skills consists of oral intake of pureed foods, and drinking from a bottle. Sleep is poor with frequent night awakenings, and he experiences jerking while falling asleep as well as seizures during sleep. He has hyperactive behavior according to his parents.

**Family 10**

He is a 5-year-old male who was born full term weighing 3824 grams (38.2%ile). Seizures began at 27 months of age of the tonic-clonic and absence types. ESES has been documented on EEG. Most recent antiepileptics include Keppra, valproate, clobazam. Brain MRI showed some nonspecific white matter changes in the frontal lobes but otherwise was read as normal. Seizures mainly occur at night, and his sleep is reported to be restless. Developmental delay was first appreciated at 7 months when he did not sit. His developmental assessments estimate him to be 2-2.5 years behind. He has ataxia and does use a few unintelligible words, but mainly communicates with pictograms and gestures. Parents report that he is hyperactive.

**Family 11**

He is an 11-year-old male who was born full term weighing 3629 grams (26.8%ile). Seizures started at 23 months old and he has tonic-clonic and absence seizures with intermittent control. An EEG at age 10.5 years demonstrated bifrontal discharges and a CSWS pattern. Brain MRI in the past was read as normal. Most recent medications include epilim, Keppra, clobazam and midozalam. Developmentally, he started walking at 2.5 years of age. He began using words and putting some together at as a 9-year-old but still is difficult to understand. He is not potty trained. His sleep is described as restless, and often he has seizures while falling asleep. Hyperactivity is not reported by parents.

**Family 12**

He is a 3-year-old male who was born full term weighing 3600 grams (25.1%ile). Seizures were diagnosed at age 2, and he has absence and atonic seizures which remain poorly controlled. EEG was read as prolonged seizures. Most recently, he was on Onfi. Developmentally, at the chronological age of 2.5 years, he functions at the 12-month-old level, babbles, and will say “mama” and “dad”. He does not use a fork or spoon and does not have a pincer grip. Ambulation is with a gait trainer and stroller. He has frequent awakenings during sleep. Hyperactivity is not reported by parents.

**Family 13**

He is a 8-year-old male who was born full term weighing 2807 grams (3.1%ile). Seizures were diagnosed at age 2 years, although earlier unrecognized seizure activity is suspected. He has tonic-clonic and drop seizures, as well as seizures characterized by mouth twitching, jerking, twitching and laughing. These seizures are difficult to control even on multiple medications (currently clobazam and epilim) and with VNS placement. His most recent EEG at age 8 was read as ESES. Brain MRI in the last year was normal. He started walking at age 2, and was able to say “mom”, but then at age 5 years he lost the ability to walk and now is nonverbal. He uses a wheelchair for ambulation, and requires G-tube feeding and assistance with all activities of daily living. Sleep duration is a maximum of six hours and of poor quality, with significant seizure activity and continuous jerking movements predominantly occurring at night. He has a diagnosis of autism and attention deficit hyperactivity disorder.
